# Genome-Wide Association Studies and QTL Mapping Reveal a New Locus Associated with Resistance to Bacterial Pustule Caused by *Xanthomonas citri* pv. *glycines* in Soybean

**DOI:** 10.3390/plants13172484

**Published:** 2024-09-05

**Authors:** Rafaella Cardoso-Sichieri, Liliane Santana Oliveira, Valéria Stefania Lopes-Caitar, Danielle Cristina Gregório da Silva, Ivani de O. N. Lopes, Marcelo Fernandes de Oliveira, Carlos Arrabal Arias, Ricardo Vilela Abdelnoor, Francismar Corrêa Marcelino-Guimarães

**Affiliations:** 1 Center for Biological Sciences, Londrina State University (UEL), Celso Garcia Cid Road, km 380, Londrina 86057-970, PR, Brazil; rafaellacardoso_@hotmail.com; 2 Department of Computer Science, Federal University of Technology of Paraná (UTFPR), Alberto Carazzai Avenue, 1640, Cornélio Procópio 86300-000, PR, Brazil; liliane.sntn@gmail.com; 3 Department of Plant Sciences, University of Tennessee, Knoxville, TN 37996, USA; valopes@gmail.com; 4 Brazilian Agricultural Research Corporation (Embrapa Soja), Carlos João Strass Road, Warta County 86085-981, PR, Brazil; danielle.gregorio-silva@embrapa.br (D.C.G.d.S.); ivani.negrao@embrapa.br (I.d.O.N.L.); marcelofernandes.oliveira@embrapa.br (M.F.d.O.); carlos.arias@embrapa.br (C.A.A.); ricardo.abdelnoor@embrapa.br (R.V.A.)

**Keywords:** GWAS, genotyping by sequencing (GBS), marker-assisted selection, recessive

## Abstract

Bacterial pustule (BP), caused by *Xanthomonas citri* pv. *glycines*, is an important disease that, under favorable conditions, can drastically affect soybean production. We performed a genome-wide association study (GWAS) with a panel containing Brazilian and American cultivars, which were screened qualitatively and quantitatively against two Brazilian *X. citri* isolates (IBS 333 and IBS 327). The panel was genotyped using a genotyping by sequencing (GBS) approach, and we identified two main new regions in soybeans associated with *X. citri* resistance on chromosomes 6 (IBS 333) and 18 (IBS 327), different from the traditional *rxp* gene located on chromosome 17. The region on chromosome 6 was also detected by QTL mapping using a biparental cross between Williams 82 (R) and PI 416937 (S), showing that Williams 82 has another recessive resistance gene besides *rxp*, which was also detected in nine BP-resistant ancestors of the Brazilian cultivars (including CNS, S-100), based on haplotype analysis. Furthermore, we identified additional SNPs in strong LD (0.8) with peak SNPs by exploring variation available in WGS (whole genome sequencing) data among 31 soybean accessions. In these regions in strong LD, two candidate resistance genes were identified (Glyma.06g311000 and Glyma.18g025100) for chromosomes 6 and 18, respectively. Therefore, our results allowed the identification of new chromosomal regions in soybeans associated with BP disease, which could be useful for marker-assisted selection and will enable a reduction in time and cost for the development of resistant cultivars.

## 1. Introduction

Bacterial pustule (BP), caused by *Xanthomonas citri* pv. *glycines* (*Xcg*; previously classified as *Xanthomonas axonopodis* pv. *glycines*) [1], is a soybean disease that affects the major soybean-producing countries in the world [2]. There are reports of severe crop damage caused by BP. In Thailand, losses due to BP were considerably high whenever the recommended variety, SJ, was grown [3]. In India, severe incidence ranging between 10.5 and 77.8% was reported to cause yield declines of up to 37.7% [4]. In such examples, losses were associated with planting susceptible varieties. In Brazil, where this disease has a widespread occurrence, being noticed in the majority of the producing states [5]), reports of damages are scarce. This occurs because farmers have been planting disease-resistant cultivars for at least the last 20 years [2].

Symptoms of BP begin with small, light green spots with raised centers, more often observed on the abaxial leaf surface. Lesions vary in size from small to large, with irregularly shaped brown areas surrounded by chlorotic halos. Generally, light-colored pustules develop in the center. Lesions can further coalesce to form large, necrotic patches. Strong yellowing of the leaf can result from heavy inoculation. Severe infections frequently result in premature defoliation, leading to a decrease in size and number of seeds. The bacteria survive through the winter on crop residues and are spread by wind or rain splashes, and they can be transmitted through contaminated seeds. Bacteria gain entrance to the plant through natural openings and wounds and need warm, moist conditions [5,6,7].

The occurrence of *Xcg* has shifted in the last decades. It has been reported in areas where it had not been previously found, such as in the north of the United States [8], and its incidence has been increasing due to global warming and the more frequent occurrence of storms [9]. Besides that, the natural variation of this bacteria is wide. Many studies have classified them into different races [10,11], with different patterns of aggressiveness [12,13,14,15]. An equivalent variety of soybean resistance genes would be useful to cope with these diverse and evolving pathogen populations.

Previous studies have established that resistance against BP is controlled by a single recessive gene, initially identified in the soybean cultivar CNS (PI 548445) [16]. This resistance gene was named *rxp* [17] and was mapped on chromosome (chr) 17 (linkage group D2) at a region of an approximate 1 Mb interval [18,19]. Subsequently, mapping studies on the Korean accession Danbaekkong narrowed the *rxp* locus to a 33 kb region, between the SNP SNUSNP17_12 and the SSR marker SNUSSR17_9 [20]. QTLs have also been identified for resistance to some *Xcg* isolates in this same material, Danbaekkong, and in another material, SS2-2, suggesting that resistance to BP is controlled by a major gene (*rxp*) and other minor genes [21,22]. For instance, the study of Zhao et al. (2022) [23], combining genetic and association mapping, identified one major QTL in chr17 (approximately in the same region as *rxp* from CNS) explaining 74.33% of the resistance and two minor QTLs, on chromosomes 5 and 17, that accounted for 7.26% and 22.26% of the total phenotypic variations, respectively. Other studies have also described a different major gene associated with BP resistance on chromosome 10, using the source of resistance PI 96188 [24,25,26,27]. In this accession, disease symptoms included only pustules resulting from the HR (hypersensitive response), without the presence of yellowish halos around the lesion, characteristic of plants that have the *rxp* resistance gene [25,26,27]. Even immune reactions to an *Xcg* isolate from India have been characterized in the soybean lines P-4-2 and P-169-3. The reaction found in P-4-2 was demonstrated to be controlled by duplicated recessive genes [26].

Resistance to BP is an essential trait for the development of Brazilian soybean cultivars. Between 2008 and 2014, an average of 16% of the lines tested by the Embrapa Breeding Program for resistance to BP were susceptible [28]. In 2008, 2009, and 2014, 33%, 27%, and 19% of the lineages were susceptible, respectively. These numbers reinforce the importance of selection for resistance to BP. However, there is scarce information on the genetic background of Brazilian lineages and also on the genomic regions responsible for the resistance of Brazilian cultivars. Considering common ancestors, it is expected that the rxp gene described in the American cultivars may have contributed to the BP resistance present in the Brazilian cultivars, since CNS is one of the main ancestors of the Brazilian soybean [29]. To date, only one study [30] has aimed to map the resistance of the Brazilian germplasm against two *Xcg* strains, one collected in Brazil (2440) and one from Sudan (2447). Besides a significant marker in Chr17 associated with resistance to the Sudanese strain, which could have been inherited from CNS, the authors found association in other chromosomal regions, mainly in chromosomes 3, 13, and 15. Despite the importance and utility of this work, only one soybean accession was completely resistant to the Brazilian *Xcg* strains, indicating that genetic resistance of Brazilian cultivars to Brazilian *Xcg* strains has not yet been completely explored.

The majority of the mapping studies available for BP resistance focus on QTL mapping (quantitative trait locus) in biparental crosses, which can limit resolution of the mapping due to the small number of recombination events that are captured in such populations, since the diversity sampled is limited to two parents per population [31]. In this context, the genome-wide association study (GWAS) is a better alternative mapping approach, since it captures the historical events of recombination that occurred during the evolution of the panel analyzed, leading to more accurate QTL positions [32,33]. The use of the GWAS approach in soybeans is well established from a variety of studies and has identified markers associated with resistance to many diseases, yield, and other traits [34,35,36,37,38,39,40]. These studies highlight the importance of GWAS as a strategy for identifying genes and regions related to agronomic characters of interest for soybean breeding. Despite the potential of GWAS to reveal regions associated with important traits, only a few studies have applied the GWAS approach to study the resistance to BP [30,41]. SNP markers associated with these regions can be useful in marker-assisted selection (MAS) to cope with the variability of strain/races of the pathogen in different geographic regions.

Thus, to better understand the genetic basis of BP resistance, we conducted a GWAS on a diverse soybean panel composed of Brazilian and American cultivars and Asian accessions against two *X. citri* isolates collected in Brazil, by analyzing both Genotyping y Sequencing (GBS) [42,43] and Illumina Infinium BeadChip SoySNP50K [44] genotypic data. We aimed with this work to identify the genomic regions underlying the resistance against bacterial pustule present in Brazilian germplasm. We also developed a segregant population to confirm one of the new loci found. We provided haplotypes that can be used as molecular markers to facilitate the selection for resistance to BP.

## 2. Results

### 2.1. Phenotypic Evaluation of Bacterial Pustule Resistance in Soybean Accessions

**Figure 1:**
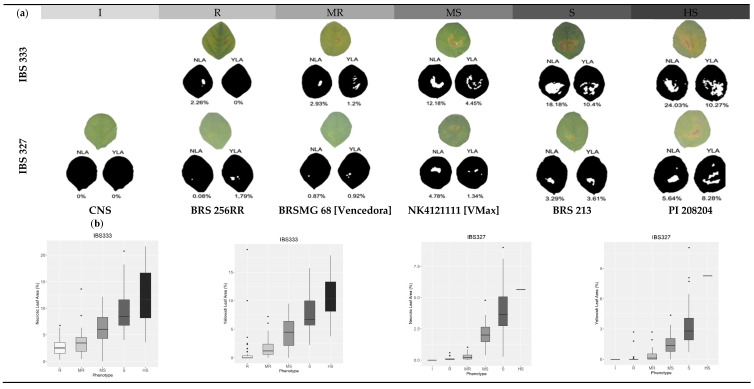
(**a**) Phenotypic reaction of six genotypes (CNS, BRS 256RR, BRSMG 68 [Vencedora], NK4121111 [VMax], BRS 213, PI 208204) to the isolates IBS 333 and IBS 327 of *Xanthomonas citri*, representing the classes (I, R, MR, MS, S, and HS), and (**b**) distributions of the quantitative phenotypes throughout the genotypes classified in each class. NLA = necrotic leaf Area; YLA = yellowish leaf area; I = immune; R = resistant; MR = moderately resistant; MS = moderately susceptible; S = susceptible; and HS = highly susceptible.

The response of the germplasms to bacterial pustule was evaluated both quantitatively and qualitatively. Quantitative assessment involved estimating the percentage of necrotic leaf area (NLA) and the percentage of yellowish leaf area (YLA). Qualitative evaluation was conducted by visually comparing the phenotype of the leaves with the disease scale outlined in [45], albeit with modifications, and assigning one of the six disease score (DS) classes (I, R, MR, MS, S, and AS) (Supplementary Table S1). Representative reactions corresponding to each class are depicted in Figure 1a, while Figure 1b illustrates the distributions of the quantitative attributes across each disease score class. Analysis of variance for the quantitative attributes NLA and YLA obtained for both isolates revealed a significant genotype effect (*p* < 0.001). Both attributes were analyzed assuming a gamma distribution for the data, as it provided a better fit than the normal distribution. The least squares mean estimators for the genotype effect were then used to conduct the GWAS, along with the disease score.

For IBS 333, NLA ranged from 0.36% to 25.61% (with an average of 9.83%), while %YLA ranged from 0% to 26.76% (with an average of 6.22%). On the other hand, the IBS 327 isolate exhibited a milder response, with %NLA ranging from 0% to 8.28% (average of 1.46%) and YLA ranging from 0% to 4.12% (average of 0.99%) (Table 1). Additionally, 34 materials (comprising 24 Brazilian, two American, and eight Asian varieties) displayed no lesions for this isolate, indicating an immune reaction. The disease scores and quantitative values for each accession for both isolates are detailed in Supplementary Table S2.

In terms of disease scores and their distributions for the 182 genotypes inoculated with IBS 333 and the 181 genotypes inoculated with IBS 327, the immune reaction was observed only for the isolate IBS 327. In total, 68 accessions (comprising 49 Brazilian, four American, and 15 Asian varieties) demonstrated simultaneous resistance to both isolates (classes 0, 1, or 2) (Supplementary Figure S1). These groups included the resistant controls Williams 82 and CNS. Similarly, 64 accessions (24 Brazilian, three American, and 37 Asian) were simultaneously susceptible to both isolates (classes 3, 4, and 5), including the susceptible control PI 416937. Only one North American accession exhibited a highly susceptible phenotype against isolate IBS 327, while for isolate IBS 333, there were 18 Brazilian, four American, and 16 Asian accessions with this phenotype (Supplementary Table S2). On the other hand, 13 accessions showed contrasting resistance responses to each of the isolates, with four and nine accessions classified as bearing some level of resistance (classes 1 and 2) exclusively to isolate IBS 333 and isolate IBS 327, respectively. These results indicate that the resistance present in the panel differs for each of the isolates.

### 2.2. Genome-Wide Association Study for Bacterial Pustule Resistance

After running the Fast-GBS pipeline [39], we identified 195,235 high-quality SNPs distributed across the 20 soybean chromosomes, averaging 9762 SNPs per chromosome. Consistent with expectations, chromosome 18, the largest chromosome in soybeans, exhibited the highest number of SNPs (14,717 SNPs). Similarly, the lowest number of SNPs was observed on chromosome 12 (2467 SNPs), the second smallest chromosome (Supplementary Table S3). Following filtering criteria (minor allele frequency—MAF ≥ 5% and heterozygosity—HET ≤ 10%), we retained 26,922 and 26,296 polymorphic SNPs in the GWAS panels for the isolates IBS 333 and IBS 327, respectively.

Principal component analysis (PCA) of both panels revealed widely dispersed accessions, indicating no distinct grouping based on their resistance profiles. Notably, a similarity between the Brazilian and American cultivars emerged, as these groups were closely aligned (Supplementary Figure S2).

**Figure 2:**
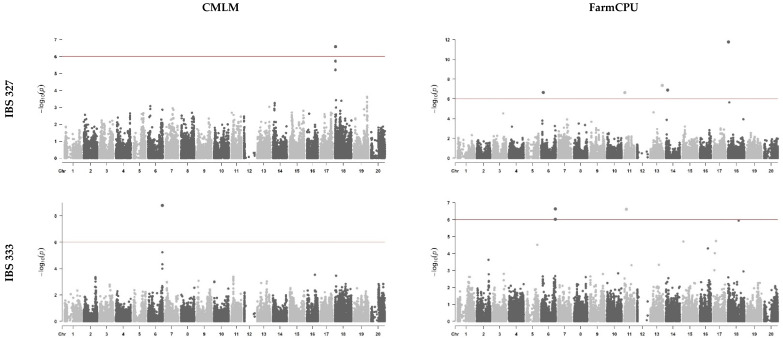
Manhattan plots showing genomic regions related with resistance to bacterial pustule, caused by *Xanthomonas citri*, identified via GWAS using qualitative (disease score) phenotyping data across 20 soybean chromosomes. Both isolates (IBS 333 and IBS 327) were included, using the entire panel. The red lines in the graphs correspond to the threshold line.

GWAS analyses for qualitative phenotyping with the IBS 333 isolate revealed a single significant marker associated with BP resistance, located at position 49,886,965 on chromosome 6. This marker was consistently detected across all models (Figure 2 and Table 2) and was also identified by the BLINK model using the quantitative attributes (NLA and YLA). Additionally, other significant SNPs were found on chromosomes 2, 3, 5, 6, 7, 11, 13, 17, and 18. These SNPs were detected using the multilocus models FarmCPU and/or BLINK, utilizing either DS or the quantitative attributes. Notably, all models for chromosome 6, position 49,886,965, exhibited an MAF of 44% and negative effects ranging from −2.2 to −0.41.

For the GWAS analyses conducted with IBS 327 data, significant SNPs were only detected using the qualitative phenotype. A significant SNP was identified on chromosome 18 at position 1,872,252, by all four models employed, exhibiting a MAF of 44% and negative effects ranging from −0.70 to −0.57. Additionally, the MLM and CMLM models identified another SNP on the same chromosome at position 1872108, with MAFs of 43% and positive effects of 0.66 (Figure 2 and Table 2). Furthermore, the FarmCPU model displayed multiple peaks for isolate IBS 333 (Figure 2), also revealing significant SNPs on chromosomes 6, 11, 13, and 14. These loci exhibited MAFs ranging from 18% to 50% and effects varying from −0.49 to 0.51.

In the analysis of subsets categorized by phenotypic responses, no regions significantly associated with BP resistance were identified for subset 1, consisting of accessions exhibiting an immune response to BP infection caused by the IBS 327 isolate (Supplementary Figure S3). Conversely, in subset 2, comprising accessions displaying HR lesions without yellowish halos around the lesion (observed exclusively with the IBS 333 isolate), the same significant marker associated with BP resistance (position 49,886,965, chromosome 6) was identified, as previously revealed (Supplementary Figure S4). Furthermore, the FarmCPU statistical model exclusively detected other allelic variations, with the corresponding SNPs listed in Table 2.

In the association analysis conducted using a subset panel and SNPs derived from SoySNP50K, an allelic variation on chromosome 6 (Chr6:49,870,244) was identified, exhibiting a significant association with resistance to BP against the IBS 333 isolate. As expected, this genomic region overlapped with the findings from the GWAS conducted using the GBS approach. However, it is worth noting that no significant association was observed within the panel against the IBS 327 isolate (Supplementary Figure S5).

### 2.3. Haplotype Analysis

**Figure 3:**
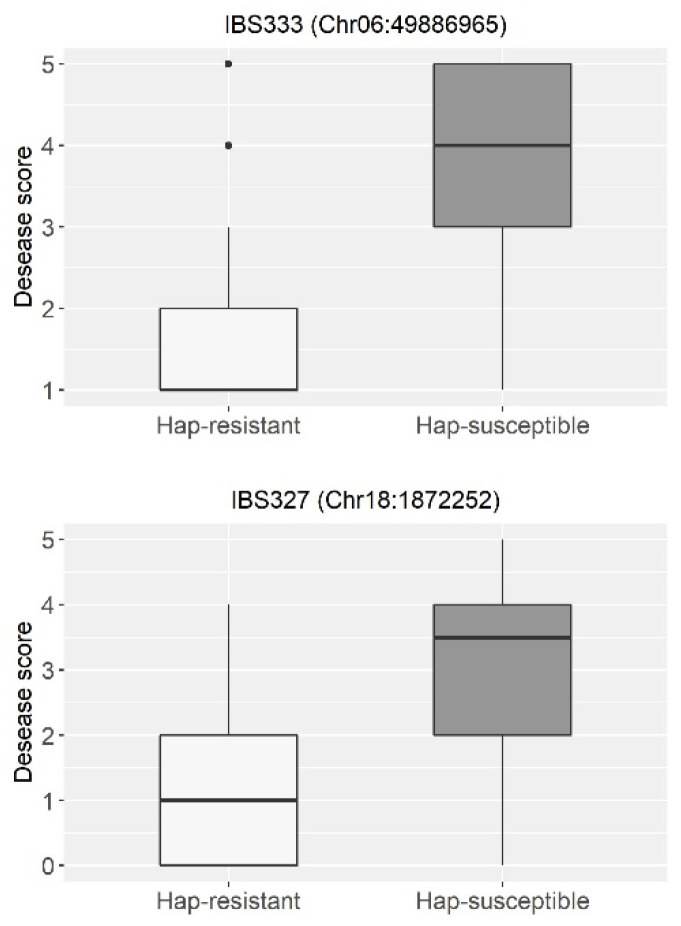
Boxplot depicting disease scores among soybean genotypes inoculated with *Xanthomonas citri*, highlighting SNP variations identified through GWAS for isolates IBS 333 and IBS 327.

For the haplotype analysis, the two main regions identified in the mapping interval for the isolates IBS 333 and IBS 327 were accessed, corresponding to chromosomes 6 and 18, respectively, and only the homozygous results were included in the analysis. Initially, considering the 173 materials assayed for IBS 333 and the peak SNP Chr6:49,886,965 (T/C), the resistant allele (T) was observed in materials classified as MR and R (genotype/phenotype agreement—GF of 86%), with the prevalent susceptible allele (C) found in accessions classified as MS, S, and HS (GF of 78%) (Figure 3, Table 3 and Table S4). Interestingly, although the susceptible allele (C) was more prevalent in susceptible materials, the resistant allele (T) was more specific to materials displaying resistant phenotypes.

Furthermore, the peak SNP Chr18:1,872,252 (T/G) identified in the mapping for IBS 327 was analyzed. Here, a prevalence of the resistant allele (T) was found in MR and R materials (76%), whereas the “G” allele was predominant in the MS, S, and HS categories, representing 71% (Figure 3, Table 3 and Table S4). Notably, the prevalence rate of the “T” allele in accessions exhibiting an immune response was even higher, with 28 out of 34 accessions presenting immune phenotyping displaying this allele (Table 3).

Among the 61 accessions resistant to both isolates, 34 individuals presented with “T” alleles for both peak SNPs (Chr06:49,886,965 and Chr18:1,872,252). Of these, 25 displayed immune resistance response for the IBS 327 isolate. Of note, no individuals classified as highly susceptible to one or both isolates showed this allele combination (Supplementary Table S5).

Finally, regarding the peak SNP identified in the SoySNP50K set (Chr06:49,870,244 A/C), most materials resistant to IBS 333 exhibit the “A” allele (59/62), while susceptible cultivars have the “C” allele (37/41) (Supplementary Table S6).

We were able to evaluate seven Brazilian cultivars ancestors, including CNS, Tokyo, and S-100, with IBS 333, and to conduct their haplotype analysis for the SoySNP50K-derived SNP. We observed a high concordance among the haplotype and phenotypic reactions. The exception was the cultivar Tokyo, which exhibited resistance to isolate IBS 333 but presented the susceptible variation (Supplementary Table S7).

### 2.4. Investigation of LD Blocks Based on WGS Data and Additional Allelic Variation

**Figure 4:**
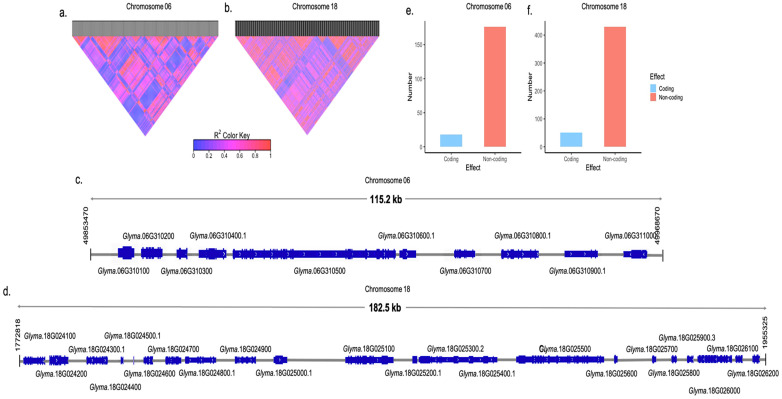
(**a**) Heatmap representing the values of r^2^ ≥ 0.8 among the 78 SNPs identified in the LD block with peak SNP Chr06:49,886,965. (**b**) Heatmap representing the values of r^2^ ≥ 0.8 among the 130 SNPs identified in the LD block with peak SNP Chr18:1,872,252. (**c**) LD region on chromosome 6 (115.2 kb) containing 10 gene models, ranging from position 49,853,470 to 49,968,670. (**d**) LD region on chromosome 18 (182.5 Kb) containing 22 gene models, ranging from position 1,772,818 to 1,955,325. (**e**) Distribution of the 78 SNPs identified in the LD block with peak SNP Chr06:49,886,965 (r^2^ ≥ 0.8) among coding and non-coding regions. (**f**) Distribution of the 130 SNPs identified in the LD block with peak SNP Chr18:1,872,252 (r^2^ ≥ 0.8) among coding and non-coding regions.

To gain insights into possible variants not detected by the GBS method and candidate genes in the region, we examined all SNPs in the LD blocks containing peak SNPs (6 and 18), using a WGS dataset composed of 31 accessions with known reactions to BP isolates. We identified 78 SNPs for chromosome 6 and 130 SNPs for chromosome 18, all with r^2^ values greater than or equal to 0.8 with the peak SNPs (Supplementary Table S8). The LD block on chromosome 6 spans 115.2 kb and contains 10 gene models (Figure 4a,c), while the LD block on chromosome 18 covers 182.5 kb and includes 22 gene models (Figure 3b,d). SNPs were identified in coding and non-coding regions (Figure 4e,f).

In chromosome 6, we identified one candidate gene containing an NB-ARC domain, characteristic of plant disease-resistance proteins (*Glyma.06g311000*). Within this gene model, four SNPs were found in coding regions, with two predicted to cause synonymous modification and two non-synonymous modifications (Supplementary Table S9). In chromosome 18, we identified one candidate gene containing LRR-LRK domains (*Glyma.18g025100*), also associated with plant disease resistance. In this gene model, seven SNPs were located in coding regions, with four in intron regions, one causing a synonymous modification, and two predicted to cause non-synonymous modifications (Supplementary Table S9).

### 2.5. QTL Mapping

The materials Williams 82 and PI 416937 exhibited highly contrasting phenotypes when assessed with IBS 333. Williams 82 displayed only HR lesions, while PI 416937 exhibited one major lesion surrounded by a yellowish halo (Supplementary Figure S6). Among the F_2_ plants, 63 were classified as susceptible, while 163 were deemed resistant, thus confirming the expected 1:3 ratio by the chi-squared test (Table 4). This suggests that resistance to IBS 333 observed in Williams 82 is controlled by a single recessive gene.

**Figure 5:**
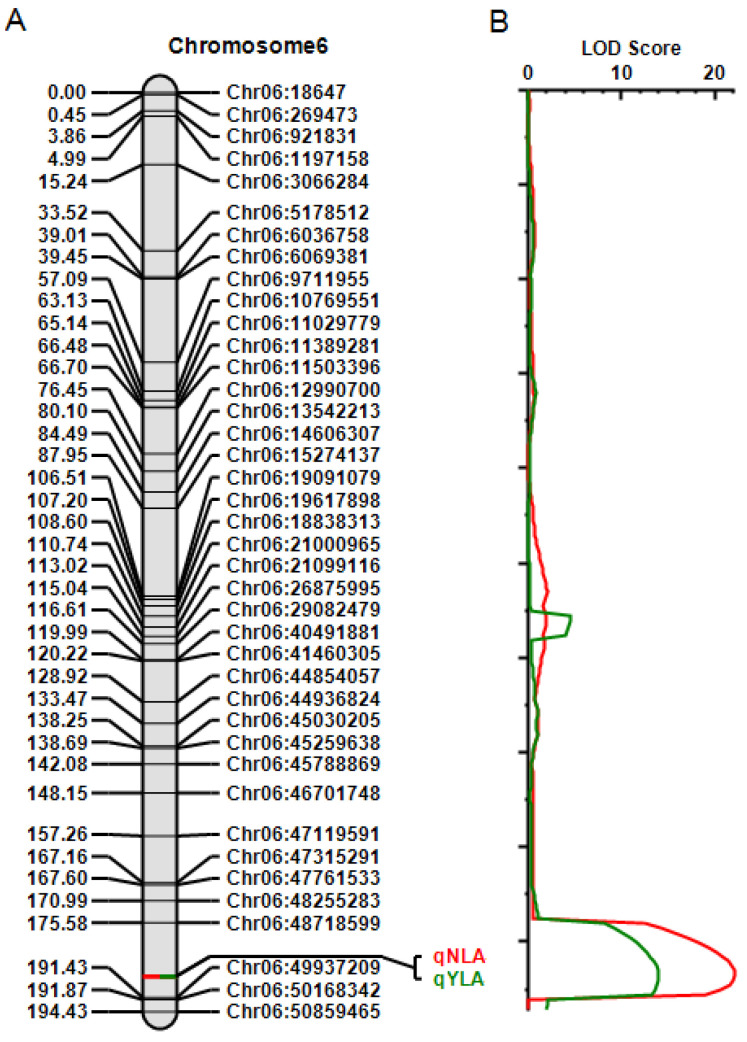
Quantitative trait loci (QTL) identified by inclusive composite interval mapping for NLA and YLA on chromosome 6 using a population of 226 F_2_ plants derived from a cross between Williams 82 (resistant to the BP isolate IBS 333) and PI 416937 (susceptible). (**A**) Genetic map of chromosome 6 with the position of the QTLs for the NLA and YLA. The genetic positions (cM) are represented on the left, with the GBS markers, corresponding to physical positions of the Williams 82 assembly (Gmax_275_Wm82.a2.v1), on the right. (**B**) LOD scores for the QTLs for NLA (red) and YLA (green). Only peaks between Chr06:48,718,599 and Chr06:49,937,209 were significant at the 5% level by the 1000-permutation test.

After applying filtering steps, 755 high-quality SNP markers that were polymorphic between the progenitors were utilized for QTL mapping. The mapping of the quantitative parameters NLA and YLA indicated two QTLs located at the same position on chromosome 6, between SNPs Chr06:48,718,599 and Chr06:49,937,209 (~1.22 Mb interval), with LOD scores of 22.03 and 13.85, respectively. This interval overlaps the GWAS region significantly associated with resistance to the same isolate (Figure 5, Supplementary Table S10).

## 3. Discussion

### 3.1. Xanthomonas citri Reactions in the GWAS Panel

Genetic resistance to BP is a highly desirable trait in soybean cultivars since it is the main control strategy for this disease. In this context, we successfully characterized the response to BP infection against two Brazilian isolates in a diverse panel composed mainly of Brazilian and American cultivars and Asian materials. These two isolates were collected in São Paulo, Brazil, in 1981 and may not represent current pathogen races in soybean fields. However, their use has successfully supported the development of resistant cultivars in Brazilian breeding programs. To date, knowledge about the bacterial variability in soybean areas is very limited in Brazil. Pustules surrounded by small yellow halos are the typical symptoms of *X. citri* infection in soybean [4,5]. When we evaluated a panel composed of 212 soybean accessions, we clearly observed that IBS 333 was more aggressive than IBS 327, confirmed by the number of materials presenting susceptible reactions, as well as by the average of the quantitative values of the lesion area (NLA and YLA). Differences in aggressiveness of *Xcg* isolates have been previously described [12,13,14,15].

When evaluated with Brazilian isolates, Williams 82 and CNS cultivars showed the “R” reaction for both isolates, displaying typical symptoms of resistance to BP and presenting only HR lesions, as previously described when they were targeted with North American isolates. Similarly, PI 416937 was classified as “AS” and “S” for isolates IBS 333 and IBS 327, respectively, showing broad susceptibility to BP [18,19].

In general, we observed that resistance to BP is prevalent in tropical adapted germplasm (49 accessions); however, 27 Brazilian and American cultivars presented a susceptibility phenotype for one or both isolates. Some of them were developed in the 80s decade, when the resistance to BP started to be introgressed in soybean germplasm in Brazil. In addition, it is also possible that pathogen variability is occurring in fields. These results highlight the importance of phenotyping with different isolates and identifying new resistant accessions to *X. citri*.

It was observed that the IBS 333 isolate caused a severe HR reaction, as revealed by the observation of the NLA parameter. Kaewnum et al. [14] mentioned that different isolates can generate variation in the induction of HR in the plant, activating a cascade of biochemical reactions, aiming to prevent the spread of the bacteria. In addition, the yellowish halo around the lesion, highlighted by the YLA parameter, is characteristic of BP susceptibility reactions, although resistant accessions can also show pustules with halos in smaller quantities [25]. Our results corroborate this study, as we found higher YLA values in susceptible materials, although we also observed these halos in some resistant materials.

We observed a considerable range of phenotypic reactions among the accessions, including accessions with immune responses, with only HR lesions, and also possessing both HR lesions and yellowish halos. According to previous reports, the *rxp* resistance gene is characterized as generating a resistance response that can include yellowish halos, while the resistance gene identified on PI 96188 is only responsible for the HR reaction, without the presence of yellowish halos around the lesion [25,26,27]. As the phenotypic reactions of our panel varied for the two isolates tested, it was not possible to clearly associate the phenotypic profile with the previous genes identified. Interestingly, Williams 82 and CNS, both previously described as containing the *rxp* gene [17,18,20], showed an R reaction when tested with both Brazilian isolates. However, here we mapped new regions associated with resistance that are not related with these described resistance genes previously mapped on soybean chromosomes 17 and 10 [18,20,25]. The materials that showed immunity and carried new resistance loci to BP are certainly valuable sources of resistance to BP in breeding programs.

### 3.2. GWAS Highlights Two Genomic Regions Associated with BP Resistance in Soybean

In this work, we used the GBS approach to identify 20,385 high-quality SNP markers, covering the 20 soybean chromosomes, a representative number of the entire genome. As expected, population structure (PCA) showed a grouping among Brazilian cultivars, which can be explained by the fact that the genetic basis of Brazilian cultivars is very narrow, most of which are derived from a few ancestors’ materials [29].

The GWAS revealed a highly significant association of resistance with PB on chromosomes 6 and 18, for the IBS 333 and IBS 327 isolates, respectively. The results indicated a strong association of an SNP on chromosome 6 in multiple analytical models for IBS 333 isolate, including SNPs derived from soySNP50k data, especially when the quantitative parameter (NLA) was used. For both association mappings, the regions associated explained around 30% of the phenotypic variation (R2 = 0.32 and 0.29 for SNPs on chromosomes 6 and 18, respectively). The allelic frequency of the SNPs was the same in both panels (0.44), demonstrating that both regions are occurring at a similar frequency in the accessions of the panel, not necessarily in the same materials.

Considering the haplotype, the SNPs detected on chromosome 6 for IBS 333 and chromosome 18 for IBS 327 were able to correctly detect the phenotype responses to the isolates. The significant markers identified in this work would be effective in tagging resistance in 65 out of the 76 accessions for IBS 333 and 71 out of the 93 for IBS 327 that were characterized as resistant, which could be used by breeders as a source of BP resistance. In addition, 34 materials, including 22 Brazilian cultivars, presented both genomic regions that may have resistance genes for both isolates and can be applied as useful sources of BP resistance for breeding in Brazil.

Surprisingly, none of the previous genetic mapping studies have mapped regions associated with resistance to *X. citri* on these chromosomes, as most were limited to mapping the resistance gene on chromosomes 17 and 10 [18,20,22,23,24,25]. Kim et al. [20] conducted fine mapping and identified two candidate genes for BP resistance on the chromosome 17 interval, identifying paralogs of these genes in six other genomic regions. Although one of these regions is located on chromosome 6, it is not the same as that found in the current study. However, most of these studies were limited to exploring QTL segregation in just one pair of accessions.

In addition, previous studies using the GWAS approach did not identify associations in the same regions found in the current study [30,41]. The first association mapping study was conducted using a panel composed only by PIs (Asian materials), which revealed regions unrelated to the panel under study [30], while the second used different isolates and accessions [41]. The composition of the panel is fundamental in identifying regions in mapping studies [36]. Our panel is diverse, consisting of Brazilian and American cultivars and Asian materials. For this reason, the identification of new regions carrying disease-resistance genes is not surprising.

Although GWAS for immune accessions did not reveal significant associations for resistance against IBS 327, 28 out of 34 immune accessions shared the resistance SNP mapped on chromosome 18 (Chr18:1872252), while 24 were also resistant to IBS 333. Interestingly, Manjaya and Pawar [26] demonstrated the segregation of duplicate recessive genes controlling resistance in the source P-2-4, which presented an immune reaction to an Indian BP isolate. We also explored a subset of our panel that presented only accessions with the HR phenotype as resistant samples. However, we did not observe a specific region associated with this type of lesion, as previously described in PI 96188 [25]. Therefore, the occurrence of HR observed in resistant accessions for IBS 333 isolate might be a characteristic reaction of the resistance conferred by the gene on chromosome 6.

Importantly, other regions, in addition to those highlighted on chromosomes 6 and 18, were also identified in this study, corroborating the hypothesis that additional minor effect genes may be involved in BP resistance. Interestingly, these regions were only identified with the FarmCPU multilocus model, which is more efficient in identifying minor effect loci [46,47]. As described in the literature, *Xcg* isolates can result in different levels of susceptibility, raising the possibility of the existence of different levels of horizontal resistance [22,27]. Depending on the isolate used, resistance to BP can be overcome, suggesting that it can be controlled by a gene complex [6,21,22]. Supporting this argument, in our panel, it was possible to observe accessions showing contrasting reactions for both isolates, possibly indicating the occurrence of additional minor loci controlling resistance. Therefore, the results of this work further support the possibility that other genes may contribute to resistance to BP.

### 3.3. New Resistance Locus to BP Confer Resistance to Tropical Adapted Soybean Cultivars and Their Ancestors

In addition to the polygenic inheritance, resistance to BP has already been described as controlled by a single and recessive gene [16]. Here, we confirm the single gene inheritance by the analysis in a biparental cross, showing that the resistance inheritance of the cultivar Williams 82 to the isolate IBS 333 is determined by a single recessive resistance gene mapped on chromosome 6, in the same region identified in the GWAS.

Among the domesticated North American soybean cultivars, field resistance to BP was initially identified in CNS, a cultivar highly resistant to the disease [16]. The resistance in this cultivar is conditioned by a single recessive gene (*rxp*) mapped on chromosome 17 [16,17,18]. CNS has been identified as the most prominent ancestor of American southern cultivars. Gizlice et al. [48] reported that 17 ancestors constituted the majority of the southern US genetic base and estimated that CNS had an average coefficient of parentage of 24.7% in public cultivars that were released between 1947 and 1988. Delannay et al. [49] reported that this cultivar was present in the pedigree in each of the 48 American southern cultivars evaluated, including Williams 82. Based on its genealogy, Williams 82 may have inherited the *rxp* gene from CNS [10,15,18,48,49].

The soybean breeding program in Brazil originated from the introgression of North American cultivars toward the end of the 60s [29,50]. The same authors described 26 soybean ancestors with significant contributions to the Brazilian soybean germplasm, including CNS. In addition to CNS, three other cultivars have made significant contributions to the Brazilian soybean germplasm: PI 548485 (Roanoke), PI 548493 (Tokyo), and PI 548488 (S-100) [29]. Thus, we also speculate that BP resistance in Brazilian tropical cultivars would be conferred by the *rxp* gene derived from American ancestors. Interestingly, the new loci on chromosome 6 identified by GWAS was also confirmed by QTL mapping in the resistance source William 82. In addition, based on haplotype analysis, we tracked the occurrence of this locus in the main Brazilian ancestors CNS and S-100.

The presence of the new gene on chromosome 6 in Williams 82 does not exclude the possibility that this cultivar also has the *rxp* gene, derived from CNS. Previous studies have shown different reactions of Williams 82 to different *Xcg* strains and races [10,11,12]. Athinuwat et al. [10] identified the variability of avirulence factors (avr) of the bacteria, demonstrating differential responses in Williams 82 to different *Xcg* isolates. In a recent work, Kang et al. [15] identified that this cultivar was resistant to different natural *Xcg* strains, suggesting that it may contain other resistance loci besides the *rxp*, corroborating our results.

### 3.4. Exploring the Regions Identified by GWAS

In order to find additional markers associated to BP resistance, we explored the LD blocks associated with the peak SNPs identified using 31 soybean accessions based on WGS data. This approach was revealed to be effective in identifying additional SNPs not detected by the GBS technique, with the same discrimination power of the significant SNPs. These results expand the possibilities of choice regarding SNP maker assays for MAS.

Considering the SNPs in complete LD, we were able to narrow them down to a region of 115.2 kb and 182.5 kb for chromosomes 6 and 18, respectively. Within these regions, we detected at least one candidate gene encoding a protein containing conserved domains presented in NLR (nucleotide-binding leucine-rich repeat) resistance (R) genes: the *Glyma.06g311000* on chromosome 6, encoding a nucleotide binding site-leucine-rich repeat (NB-ARC domain) and the *Glyma.18g025100* encoding an LRR (Leucine-rich repeat) N-terminal domain on chromosome 18. The NLR genes have already been functionally characterized as inducing hypersensitivity responses to pathogen attacks and presenting a dominant genetic inheritance [51,52,53,54,55].

Like other plant pathogens, *Xcg* delivers effector proteins into the cells of its host, aiding colonization and contributing to disease development. To date, all examined *Xcg* strains encode transcription activator-like effectors (TALEs) [10,11,56,57], which are delivered into the host nucleus and activate host genes by binding to effector-specific promoter sequences. These genes are key host susceptibility genes, essential for bacteria to cause disease. The allelic variation that prevents TALE binding and activation of an important susceptibility gene can confer genetically recessive resistance to the pathogen through loss of susceptibility [58].

Depending on the host genotype, some TALEs may also trigger plant defense by activating executor resistance genes [59], which are, in this case, genetically dominant. In the Xag-soybean pathosystem, some TALEs that trigger resistance have been described, but the corresponding resistance genes have not been identified [10,11]. Based on this and considering the recessive nature of the resistance conferred by the chromosome 6 locus, the gene mapped here might also be an allele of a TALE-targeted susceptibility gene.

For the *rxp* locus in chromosome 17, a fine mapping study defined a 33 kb interval and identified genes encoding a DNA polymerase and other potential candidate genes encoding a membrane protein and a zinc finger (C3H4-type RING finger) family protein [20]. Usually, these family members contain predicted transmembrane regions like Mildew Resistance Locus (MLO) [60], which is a well-known previously described recessive resistance gene. In addition to MLO, only a few recessive genes conferring resistance, including RRS1-R and Xa5, have been identified in plants [61,62]. Different from plant dominant resistance genes, recessive genes have various structures, so their functions can vary and can be explained by the genes that may be required for pathogen growth or reproduction. For example, the Xa5 gene, a bacterial blight resistance gene in rice, encodes the gamma subunit of transcription factor IIA. Two missense mutations cause nucleotide substitutions, leading to the resistance phenotype [61]. On the other hand, MLO encodes a membrane protein containing seven transmembrane domains, and its function seems to be a negative regulator of the defense against powdery mildew fungus in barley [60]. Finally, the RRS1-R gene from *Arabidopsis* gives resistance to bacterial wilt and encodes an NBS-LRR protein [62]. In rice, the translation initiation factors, eIF4E and eIF4G, are responsible for resistance to rice yellow mottle virus and are recessive [63].

Considering the previously characterized recessive genes, we explored the functional annotation of the genes on the chromosome 6 locus, and we could extend our list of candidate genes including a zinc ion binding protein (*Glyma.06g310400*), and for chromosome 18, we could add a basic helix-loop-helix (bHLH) DNA-binding and a eukaryotic translation initiation factor 2 subunit 1, encoded by soybean genes *Glyma.18g024800* and *Glyma.18g025100*. All predicted gene models are interesting targets to be characterized in future functional studies aiming at understanding their effects on soybean plants during *X. citri* infection.

## 4. Materials and Methods

### 4.1. GWAS Plant Materials and QTL Mapping Population

A panel of 212 soybean accessions composed of 125 Brazilian cultivars, 14 American cultivars, and 75 Asian materials (PIs) (Supplementary Table S2), with variable virulence profiles to BP isolates, was investigated. The Brazilian cultivars were chosen based on previous phenotyping data at Embrapa Soja (data not shown), while the foreign materials were chosen based on phenotypic information available in the Germplasm Resources Information Network (GRIN). The accessions Williams 82 and CNS were used as resistant controls [10,12,48], and PI 416937 [18] was used as a susceptible control. The seeds for all accessions were obtained from the Embrapa Soybean Germplasm Bank, Londrina (PR, Brazil).

A biparental F_2_ population of 226 individuals was developed from the cross between Williams 82 (resistant) and PI 416937 (susceptible) for validation of a resistant locus identified by GWAS for resistance to the BP isolate IBS 333. Williams 82 was one of the varieties resistant to this isolate and has abundant genomic information available.

### 4.2. Phenotypic Evaluation for Bacterial Pustule

The experiments for disease evaluation were conducted in a greenhouse at Embrapa Soja, Londrina (PR, Brazil), from July to October 2019 for the GWAS (one experiment for each isolate) and from March to June 2020 for the biparental population. The experimental design was completely randomized, with three replicates, including three plants in each pot, in both experiments. Greenhouse conditions at this stage included average temperatures of ±28 °C with humidity in the range of 90–100%.

The GWAS panel was independently evaluated with two Brazilian *Xcg* isolates obtained from Instituto Biológico de São Paulo, collected in Brazil in 1981, designated as IBS 333 and IBS 327 (NCPPB 3659 and 3658, respectively). From the 212 soybean accessions selected for study, 181 were analyzed for the isolate IBS 333, and 182 were analyzed for IBS 327 (Supplementary Table S2). The whole biparental population was evaluated with the IBS 333 isolate.

The inoculum preparation, greenhouse conditions, and inoculation and evaluation methods were the same for the two isolates and for both the panel and the biparental population. Bacteria were grown in TSB medium (tryptic soy broth) and incubated at 28 °C for 48 h. Subsequently, the suspension was transferred to Petri dishes containing NA medium (nutrient agar) and incubated in a growth chamber at 28 °C for 48 h. The suspension for inoculation was produced by scraping the Petri dishes containing microorganisms and mixing them with distilled water and NaCl (0.05%), obtaining a concentration of 10^8^ CFU/mL (OD_600_ of 0.3).

Twenty-four hours before inoculation, plants were covered with plastic bags, and the greenhouse was maintained at high humidity (100%), with average temperatures of ±28 °C. The third trifoliums of four-week-old soybean plants (V3 growth stage, when the third trifoliate has fully emerged) were inoculated by spraying a bacterial suspension on both sides of the leaves using an atomizer. The sprays were able to be done with equal pressure and distance from the leaf surface.

Qualitative and quantitative evaluations were performed independently. For the qualitative visual classification of reactions, a disease scale proposed by [45], with some modifications (Supplementary Table S1), was applied. The leaves were phenotyped for each isolate seven days after inoculation. The plants were scored using a disease score (DS) scale ranging from 0 to 5, according to the level of disease: 0—immune (I); 1—resistant (R); 2—moderately resistant (MR); 3—moderately susceptible (MS); 4—susceptible (S); 5—highly susceptible (HS) (Supplementary Table S2).

For quantitative evaluation, one leaf from the third trifolium of each replicate was collected and stored in a freezer at −20 °C. A Nikon D5000 camera (Tokyo, Japan) was used to photograph one leaflet from each plant. Subsequently, the images of the lesions were individually analyzed using ImageJ software (Madison, WI, USA) [64], utilizing a plug-in developed by Renove AgroPesquisa (unpublished), with modifications. To estimate the percentage of leaf area affected, two filters were used, one for the color yellow and the other for brown. The regions considered in each filter were not mutually exclusive, that is, they overlapped in some of the observations. Two parameters were considered: (1) percentage of necrotic leaf area (NLA), which considered only the necrotic or HR region (brown filter), and (2) percentage of yellowish leaf area (YLA), which considered solely the yellowish area of the lesion (yellow filter). For both attributes, the values were adjusted based on the entire leaf lesion area. Plants with quantitative parameter results equal to zero were classified as immune. These quantitative data were subjected to the analysis of variance (ANOVA), using the procedure glimmix from the SAS SAS/STAT software, Version 9.4^®^, Copyright© 2016 SAS Institute Inc. (Cary, NC, USA). These analyses were performed assuming the completely randomized experiment model, having genotypes as a fixed effect and repetition as a random of the residual type. The distribution of the data was tested to determine whether it followed a normal or gamma distribution, with gamma being chosen as it provided a better fit. The least squares mean estimators for each genotype were then obtained using the ilink option from the lsmeans statement.

### 4.3. DNA Extraction, GBS Library Preparation, and SNP Calling for GWAS

Leaf samples for each GWAS-soybean accession and biparental mapping population were collected and stored at −80 °C. The DNA purification was performed using the DNeasy Plant Mini Kit (Qiagen, Inc., Valencia, CA, USA), from 100 mg of young leaf tissue (14 days after germination in the V1 stage), following the manufacturer’s protocol. Subsequently, DNA was quantified using a Nanodrop 8000 spectrophotometer (Thermo Scientific; Wilmington, DE, USA) and diluted to 10 ng/µL.

The GBS libraries for the GWAS panel were constructed according to the protocol proposed by Elshire et al. [42], modified by Sonah et al. [39]. Briefly, DNA was digested using the *Ape*KI restriction enzyme, ligated to specific adapters and barcode adapters. The GBS libraries were then sequenced using an Ion Torrent sequencer (Thermo Scientific; Wilmington, DE, USA) at the IBIS—Institute of Integrative Biology and Systems, Université Laval, Quebec City, QC, Canada. Data were processed with the Fast-GBS pipeline [65] using the Williams 82 assembly (Gmax_275_Wm82.a2.v1) [66] as a reference. Subsequently, all heterozygous genotypes were removed and replaced with missing data, and only SNPs with less than 80% missing data (call rate = 80%) and minimal allelic frequency (MAF) ≥ 1% were kept.

Imputation of missing data was performed using Beagle software v.4.1 internally in the panel [67]. Before conducting the GWAS analyses, the resulting SNPs were filtered by TASSEL 5.0 software [68]. SNPs with MAF ≥ 5% were maintained, while SNPs with heterozygosity levels above 10% (HET ≥ 10%) were eliminated, and the resulting set of SNPs was used for GWAS.

### 4.4. Panels Composition and SoySNP50K Analysis

The whole panel of soybean accessions was initially tested (212 accessions) in GWAS; 181 were analyzed for the isolate IBS 333, and 182 were analyzed for IBS 327. Furthermore, two subsets were generated based on the type of phenotype reactions: (1) accessions containing only immune reactions (34 accessions) and (2) accessions with phenotypes characterized by HR lesions without the presence of yellowish halos (38 accessions). Both subsets were joined with materials presenting susceptible reactions.

A similar analysis using both isolates was conducted with a subset of the panel composed of 117 cultivars (115 Brazilian, one American, and one Asian), with SNPs derived from the Illumina Infinium BeadChip SoySNP50K (Illumina Inc., San Diego, CA, USA) [44]. Those 117 accessions have been analyzed previously as part of a study of the polymorphism of the Brazilian germplasm (data not published). Briefly, the DNA of the accessions was submitted for analysis at Beltsville Agricultural Research Center, USDA ARS, Beltsville, MD, USA, with the Illumina platform (Illumina Inc., San Diego, CA, USA). After filtering with MAF ≥ 5%, the hybridization data generated approximately 20,000 SNPs.

### 4.5. Population Structure and Association Mapping

The GWAS analysis was performed with the GAPIT V3 (Genome Association and Prediction Integrated Tool) R package [69], using the compressed mixed linear models (MLM and cMLM—unilocus), the fixed and random model circulating probability unification (FarmCPU), and Bayesian information and linkage disequilibrium iteratively nested keyway (BLINK) (Huang et al., 2018) (multilocus) models [70,71]. A principal component analysis (PCA) was conducted, adapting it to each panel composition. The relatedness between individuals was estimated with the VanRaden kinship matrix. The first three PCs were included as covariates in the model to account for population structure.

Only SNPs with an FDR-adjusted *p*-value < 0.001 were considered significant. The significant SNPs were confirmed by analyzing whole genome sequencing data from cultivars BRS 218 [Nina] and BRS 257 using IGV 2.9.4 software [72]. For the further analysis of Linkage disequilibrium detection and haplotype and the search for candidate gene annotations, only the peak SNPs mapped in different models and harboring high levels of MAF were used.

### 4.6. Linkage Disequilibrium Detection, Haplotype Analysis, and Candidate Gene Annotations

To identify additional allelic variation in linkage disequilibrium (LD) regions of the chromosomes identified in the GWAS, 31 accessions with whole-genome sequencing (WGS) data and phenotypic information were examined, comprising 28 Brazilian, one American (CNS), and two Asian materials (Supplementary Table S2). The target regions were extracted, and the LD was estimated pairwise between the peak SNPs and the SNPs from WGS using squared allelic frequency correlations (r^2^) with PLINK software 2.0 [73]. The result was plotted using the “LDheatmap” R package 1.0-6 [74]. Only SNPs with r^2^ ≥ 0.8 with the peak SNPs were used for haplotype analysis. Predictions of SNP effects were performed using SnpEff 5.0 [75].

In addition, the SoySNP50K polymorphism data of 26 accessions comprising the principal ancestors of North American and Brazilian cultivars [29,48], including the four most important ancestors of Brazilian soybean germplasm, CNS, S-100, Roanoke, and Tokyo, were added to the haplotype analysis. Annotation of candidate genes in the LD blocks was conducted using Phytozome (https://phytozome.jgi.doe.gov/pz/portal.html, accessed on 21 May 2021) and Soybase (https://www.soybase.org/, accessed on 21 May 2021) and confirmed by Uniprot (https://www.uniprot.org/, accessed on 21 May 2021).

### 4.7. Inheritance Analysis and QTL MAPPING

To determine the inheritance of the resistance, the segregation of the resistance locus was analyzed in the F_2_ population according to a goodness-of-fit to the theoretical ratios in the Chi-square (χ^2^) test. For this purpose, we used the results of the visual evaluation of the F_2_, combining individuals classified as I, R, and MR to form the resistant class and individuals classified as S, MS, and AS to form the susceptible class.

DNA extraction of the 226 F2 individuals was performed as described previously. Genotyping of the F_2_ population was performed by the GBS approach similarly to the previous description, but using the combination of *Pst*/*Msp*I restriction enzymes. After sequencing, the data were processed with the Fast-GBS pipeline [65], using the reference genome (Gmax_275_Wm82.a2.v1). Variants were removed if (1) the mapping quality (MQ) score was < 30, (2) SNPs showed read depth lower than 5, being replaced with missing data; (3) SNPs showed missing data values higher than 80%; and (4) the heterozygosity was >60%. Imputation of the residual missing data was performed by Beagle v.4.1 [67]. Finally, only SNPs with a minor allele frequency of >30% were used.

We used the QTL IciMapping 4.1.0.0 software [76] for QTL mapping. Redundant SNPs showing identical segregation patterns were grouped in the same bin loci, and linkage groups (LGs) were assembled based on physical position, with markers ordered by input and position optimized by rippling. The Kosambi mapping function was used to convert the recombination frequency into centimorgans. QTL analysis was performed using the inclusive composite interval mapping (ICIM) method, the threshold to declare a significant QTL was based on 1000 permutations, and the type 1 error equals 0.05.

## 5. Conclusions

Our study identified new regions associated with resistance to *Xanthomonas citri* pv. *Glycine* on soybean chromosomes 6 and 18 and characterized their occurrence in Brazilian soybean germplasm and its ancestors. This work highlights the presence of two new major loci, distinct from those previously mapped. The region on chromosome 6, validated by QTL mapping in Williams 82, demonstrates that this cultivar has another resistance gene in addition to *rxp*. In addition, resistance haplotypes were shared by most genotypes and could be applied in marker-assisted selection. Furthermore, we identified resistance candidate genes associated with this disease that could be better investigated in the future by means of functional studies. These new resistance loci identified will be useful in breeding programs as an alternative to cope with new variants of the bacteria that can be selected in fields and can be explored in marker-assisted selection.

## Figures and Tables

**Table 1:** Phenotypic distributions of the accessions for the GWAS studies, according to their reaction to the isolates IBS 333 and IBS 327 inferred from a disease scale [15]. I = immune; R = resistant; MR = moderately resistant; MS = moderately susceptible; S = susceptible; and HS = highly susceptible.

Disease Score	Class	IBS 333		IBS 327	
		Number of Accessions	%	Number of Accessions	%
0	I	0	0	34	18.8
1	R	64	35.2	30	16.6
2	MR	28	15.4	35	19.3
3	MS	17	9.3	25	13.8
4	S	35	19.2	56	30.9
5	HS	38	20.9	1	0.6
Total	-	182	100%	181	100%

**Table 2:** SNPs significantly associated with resistance to bacterial pustule, caused by *Xanthomonas citri*, in isolates IBS 327 and IBS 333, tested using statistical models for qualitative (DS) and quantitative (%NLA and %YLA) phenotypes (traits). SNP = single polymorphism nucleotide; Chr = chromosome; MLM, CMLM, FarmCPU and Blink are the models applied in GWAS analysis (see Section 4); DS = disease score; %NLA = percentage of necrotic leaf area; %YLA = percentage of yellowish leaf area; FDR = false discovery rate adjusted by the H and B method; *p* = *p*-value; effect = marker effect for the model; MAF = minor allele frequency.

ISOLATE	SNP			MLM				CMLM				FarmCPU				BLINK			
	Chr	Position	Trait	*p*	FDR	Effect	MAF	*p*	FDR	Effect	MAF	*p*	FDR	Effect	MAF	*p*	FDR	Effect	MAF
IBS 327	6	7,549,194	DS	-	-	-	-	-	-	-	-	2 × 10^−7^	1.2 × 10^−3^	−0.39	0.35	-	-	-	-
	11	1,564,029	DS	-	-	-	-	-	-	-	-	2 × 10^−7^	1.2 × 10^−3^	−0.49	0.18	-	-	-	-
	13	42,076,334	DS	-	-	-	-	-	-	-	-	4 × 10^−8^	5.6 × 10^−4^	0.43	0.50	-	-	-	-
	14	5,884,688	DS	-	-	-	-	-	-	-	-	1 × 10^−7^	1.1 × 10^−3^	0.51	0.20	-	-	-	-
	18	1,872,108	DS	2 × 10^−6^	1.3 × 10^−2^	0.66	0.43	2 × 10^−6^	1.3 × 10^−2^	0.66	0.43	-	-	-	-	-	-	-	-
		1,872,252	DS	3 × 10^−7^	6.8 × 10^−3^	−0.70	0.44	3 × 10^−7^	6.8 × 10^−3^	−0.70	0.44	2 × 10^−12^	4.5 × 10^−8^	−0.57	0.44	1 × 10^−11^	2.6 × 10^−7^	−0.69	0.44
IBS 333	2	40,793,724	%NLA	-	-	-	-	-	-	-	-	-	-	-	-	4 × 10^−7^	2.9 × 10^−3^	3.49	0.04
	3	39,054,333	%NLA	-	-	-	-	-	-	-	-	5 × 10^−8^	6.5 × 10^−4^	−3.17	0.05	-	-	-	-
	5	32,140,407	%NLA	-	-	-	-	-	-	-	-	1 × 10^−6^	7.6 × 10^−3^	−2.30	0.08	5 × 10^−10^	1.3 × 10^−5^	−3.70	0.08
	6	49,886,965	DS	2 × 10^−9^	4.4 × 10^−5^	−0.82	0.44	2 × 10^−9^	4.4 × 10^−5^	−0.82	0.44	2 × 10^−7^	3.3 × 10^−3^	−0.44	0.44	6 × 10^−20^	1.7 × 10^−15^	−0.93	0.44
			%NLA	-	-	-	-	-	-	-	-	-	-	-	-	7 × 10^−8^	8.8 × 10^−4^	−1.60	0.44
			%YLA	-	-	-	-	-	-	-	-	-	-	-	-	7 × 10^−11^	1.8 × 10^−6^	−2.20	0.44
	6	50,029,391	DS	-	-	-	-	-	-	-	-	9 × 10^−7^	7.7 × 10^−3^	−0.47	0.19	-	-	-	-
	6	50,048,102	DS	-	-	-	-	-	-	-	-	-	-	-	-	3 × 10^−10^	4.2 × 10^−6^	0.86	0.13
	7	25,379,321	%YLA	-	-	-	-	-	-	-	-	2 × 10^−6^	2.4 × 10^−2^	−1.18	0.18	−	-	-	-
	11	6,791,972	DS	-	-	-	-	-	-	-	-	2 × 10^−7^	3.3 × 10^−3^	0.37	0.41	-	-	-	-
		7,274,359	%NLA	-	-	-	-	-	-	-	-	6 × 10^−11^	1.5 × 10^−6^	−3.89	0.06	4 × 10^−7^	2.9 × 10^−3^	−3.60	0.06
			%YLA	-	-	-	-	-	-	-	-	-	-	-	-	1 × 10^−7^	1.8 × 10^−3^	−4.14	0.06
	13	41,078,868	%NLA	-	-	-	-	-	-	-	-	2 × 10^−7^	1.8 × 10^−3^	2.27	0.07	-	-	-	-
	17	37,084,674	%NLA	-	-	-	-	-	-	-	-	1 × 10^−6^	6.7 × 10^−3^	1.15	0.27	-	-	-	-
	18	16,111,543	%NLA	-	-	-	-	-	-	-	-	-	-	-	-	9 × 10^−7^	5.0 × 10^−3^	1.50	0.22
		16,926,588	%YLA	-	-	-	-	-	-	-	-	9 × 10^−8^	2.5 × 10^−3^	−1.78	0.24	-	-	-	-
		37,488,371	DS	-	-	-	-	-	-	-	-	1 × 10^−6^	7.7 × 10^−3^	0.48	0.18	-	-	-	-

**Table 3:** Distribution of resistance/susceptibility and genotype-phenotype agreement (GF) of SNPs identified in the mapping for isolates IBS 333 and IBS 327. Heterozygous genotypes were not included in this analysis. I = immune; R = resistant; MR = moderately resistant; MS = moderately susceptible; S = susceptible; HS = highly susceptible; N = number of genotypes; GF = genotype/phenotype agreement.

Haplotype ID	Positions in the Soybean Genome	I/R/MR	MS/S/HS	N	GF
	Chr06:49,886,965 (T/C)	IBS 333		173	
Hap-resistant	T	65	11	76	86%
Hap-susceptible	C	21	76	97	78%
	Chr11:6,791,972 (A/G)			173	
Hap-resistant	A	48	23	71	68%
Hap-susceptible	G	37	65	102	64%
	Chr13:42,076,334 (A/G)	IBS 327		165	
Hap-resistant	A	59	24	83	71%
Hap-susceptible	G	30	52	82	63%
	Chr18:1,872,108 (A/G)			164	
Hap-resistant	A	69	25	94	73%
Hap-susceptible	G	23	47	70	67%
	Chr18:1,872,252 (T/G)			163	
Hap-resistant	T	71	22	93	76%
Hap-susceptible	G	20	50	70	71%

**Table 4:** Reaction to bacterial pustule isolate IBS 333 in F_2_ soybean population derived from the cross between Williams 82 and PI 416937. Susceptible phenotype comprises the reactions MS, S, and AS. Resistant phenotype comprises R and MR. χ^2^ = chi-square distribution.

	Observed		Expected			
Plant Material	R	S	R	S	χ^2^	*p*-Value
Williams 82	2	-				
PI 416937	-	2				
F_2_	63	163	56.5	169.5	0.9971	0.318 ^ns^

^ns^ = non-significant at 0.05 probability level.

## Data Availability

All phenotypic data are provided in Supplementary Data. GBS data and GAPIT code for running GWAS are available through direct contact to the corresponding author Dra. Francismar C. Marcelino-Guimarães by email: francismar.marcelino@embrapa.br.

## Supplementary Materials

The following supporting information can be downloaded at https://www.mdpi.com/article/10.3390/plants13172484/s1: Figure S1. Venn diagram representing the resistant/susceptible materials for both isolates (IBS 333 and IBS 327) and the resistant materials exclusively to each isolate. Figure S2. 2D scatter plot (PC1 x PC2) of principal component analysis (PCA), showing the wide distribution of accessions for the total panel of IBS 333 and IBS 327 isolates. Figure S3. Genomic regions related to resistance to *X. citri* identified by GWAS using qualitative phenotyping data. It was considered subset 1, containing just accessions that have immune responses to BP for isolate IBS 327 (129 materials). Figure S4. Genomic regions related to resistance to *X. citri* identified by GWAS using qualitative phenotyping data. It was considered subset 2, containing just accessions that have HR responses to BP for isolate IBS 333 (132 materials) Figure S5. Genomic regions related to resistance to *X. citri* identified by GWAS using qualitative phenotyping data with a subset panel and SNPs derivate from SoySNP50K (117 materials). It was considered both isolates (IBS 333 and IBS 327). Figure S6. Parentals Williams 82 (R) and PI 416937 (S) showing a high contrast phenotype when evaluated with IBS 333. Table S1. Visual rating of bacterial blight for infection with IBS 327 and 333. Table S2. List of the materials used in this study and their respective qualitative and quantitative phenotyping results for IBS 333 and IBS 327 isolates. In quantitative phenotyping, two criteria were evaluated: % NLA (necrotic leaf area) and % YLA (yellowish leaf area). Table S3. Relationship among size of the 20 chromosomes of *Glycine max* and the number of SNPs identified in GBS. Information about chromosome sizes was taken from SoyBase Browser: version Glyma.Wm82.a2 (Gmax2.0) (https://www.soybase.org, accessed on 13 June 2021). Table S4. Number of materials that have the haplotype of resistance/susceptibility to SNPs Chr06:49886965 (T/C) and Chr18:1872252 (T/G), per phenotypic classes, identified in the mapping for the isolate IBS 333 and IBS 327, respectively. Heterozygous materials and those with contrasting phenotypes were excluded from the analysis. Table S5. Information of the peak SNPs in the panel of 212 materials used for the GWAS and their phenotypic reactions to Xanthomonas citri for both isolates used (IBS 333 and IBS 327). Table S6. List of the 117 accessions genotyped with BeadChip SoySNP50K that were phenotyped with both isolates (IBS 333 and IBS 327) in the panel used in this study and their respective profile for peak SNP identified in the GWAS for this panel (Chr06:49870244). The cells highlighted in gray correspond to materials that were excluded from the analysis because of non-germination. Table S7. Peak SNP analysis identified from SoySNP50K (Chr06:49870244 A/C) for Brazilian ancestral cultivars and resistance sources of BP and their respective phenotypes. Table S8. Haplotype from SNPs detected by GWAS in chromosomes 6 and 18 based on WGS data from 28 Brazilian cultivars. Table S9. Gene annotation and SNP effect. Table S10. Quantitative trait loci for the resistance to the bacterial pustule isolate IBS 333 in a F2 population derived from the cross between Williams 82 (R) and PI 416937 (S) using the ICIM approach.
