# Supplementary material for: Genome-Wide Association Studies and QTL Mapping Reveal a New Locus Associated with Resistance to Bacterial Pustule Caused by Xanthomonas citri pv. glycines in Soybean
Source: Plants (Basel). 2024 Sep 5;13(17):2484. doi: 10.3390/plants13172484 (PMC11397087; doi:10.3390/plants13172484)
Supplement: Supplementary file 1 [file plants-13-02484-s001.zip › Supplementary Figure S3.pdf]

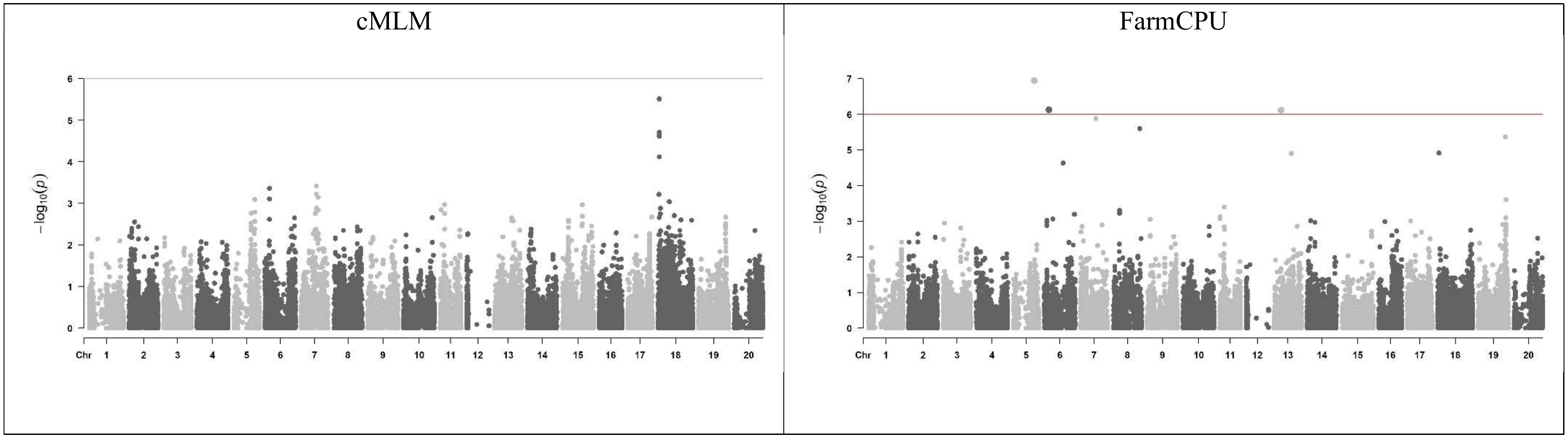

Supplementary Figure S3. Genomic regions associated with resistance to *Xanthomonas citri* identified through GWAS using qualitative phenotyping data. This analysis was conducted on genotypes exhibiting immune or susceptible responses to BP for isolate IBS 327, comprising 116 materials.
