# Supplementary material for: Genome-Wide Association Studies and QTL Mapping Reveal a New Locus Associated with Resistance to Bacterial Pustule Caused by Xanthomonas citri pv. glycines in Soybean
Source: Plants (Basel). 2024 Sep 5;13(17):2484. doi: 10.3390/plants13172484 (PMC11397087; doi:10.3390/plants13172484)
Supplement: Supplementary file 1 [file plants-13-02484-s001.zip › Supplementary Table S10_QTL mapping.pdf]

Supplementary Table S10. Quantitative trait loci for the resistance to the bacterial pustule isolate IBS 333 in a F_2 population derived from the cross between Williams 82 (R) and PI 416937 (S).

		Flanking SNPs - Positions					
QTL	Chr[1]	Genetic Interval[2]	Physical Interval[3]	LOD[4]	PVE[5]	ADD[6]	DOM[7]
NLA	6	2.175 - 2.235	48,718,599-49,937,209	22.03	31.44	2.63	-1.81
YLA	6	2.165 - 2.235	48,718,599-49,937,209	13.85	31.60	1.35	-0.95

[1]Chromosome; [2]Genetic interval in centimorgan - cM; [3]Positions according to soybean reference genome (Wm82.a2.v1); [4]LOD (logarithm of the odds) score; [5]Percent of variation explained; [6]Additive effect; [7]Dominance effect.
