# Supplementary material for: Genome-Wide Association Studies and QTL Mapping Reveal a New Locus Associated with Resistance to Bacterial Pustule Caused by Xanthomonas citri pv. glycines in Soybean
Source: Plants (Basel). 2024 Sep 5;13(17):2484. doi: 10.3390/plants13172484 (PMC11397087; doi:10.3390/plants13172484)
Supplement: Supplementary file 1 [file plants-13-02484-s001.zip › Supplementary Table S1_Visual rating of bacterial blight.pdf]

Supplementary Table S1. Visual rating of bacterial blight for infection with IBS 327 and 333.

		Symptom description	
Rating	Class	IBS 327 infection	IBS 333 infection
0	Immune (I)	No visible leaf spots	-
1	Resistant (R)	A trace of disease visible as a few spots on occasional leaves	Scattered spots covering 1-2% of the leaf area
2	Moderately Resistant (MR)	Scattered spots covering 1-2% of the leaf area	Coalescent spots covering 2-5% of the leaf area
3	Moderately Susceptible (MS)	Coalescent spots covering 2-5% of the leaf area, with occasional yellowing	Small patches covering 5-10% of the leaf area, with occasional yellowing
4	Susceptible (S)	Small patches covering 5-10% of the leaf area, with occasional yellowing	Medium size patches covering 10-25% of the leaf area, often acompanied by yellowing
5	Highly Susceptible (HS)	Medium size patches covering more than 10% of the area, often acompanied by yellowing	Large patches covering more than 25% of the area, generally acompanied by widespread yellowing
