# Supplementary material for: Genome-Wide Association Studies and QTL Mapping Reveal a New Locus Associated with Resistance to Bacterial Pustule Caused by Xanthomonas citri pv. glycines in Soybean
Source: Plants (Basel). 2024 Sep 5;13(17):2484. doi: 10.3390/plants13172484 (PMC11397087; doi:10.3390/plants13172484)
Supplement: Supplementary file 1 [file plants-13-02484-s001.zip › Supplementary Table S3_SNPs distribution on Chr.pdf]

Supplementary Table S3 Relationship among size of the 20 chromosomes of *Glycine max* and the number of SNPs identified in GBS. Information about chromosome sizes was taken from SoyBase Browser: version Glyma.Wm82.a2 (Gmax2.0) (https://www.soybase.org).

Chromossome	Chromossome Size (mb)	Number of SNPs
Chr01	56.83	9844
Chr02	48.58	9447
Chr03	45.78	10312
Chr04	52.39	11197
Chr05	51.42	7828
Chr06	51.42	11284
Chr07	44.63	8780
Chr08	47.84	9607
Chr09	50.19	9370
Chr10	51.57	10154
Chr11	34.77	7269
Chr12	40.09	2467
Chr13	45.87	10487
Chr14	49.04	10127
Chr15	51.76	11483
Chr16	37.89	9621
Chr17	41.64	9348
Chr18	58.02	14717
Chr19	50.75	9772
Chr20	47.9	10040
