# Supplementary material for: Genome-Wide Association Studies and QTL Mapping Reveal a New Locus Associated with Resistance to Bacterial Pustule Caused by Xanthomonas citri pv. glycines in Soybean
Source: Plants (Basel). 2024 Sep 5;13(17):2484. doi: 10.3390/plants13172484 (PMC11397087; doi:10.3390/plants13172484)
Supplement: Supplementary file 1 [file plants-13-02484-s001.zip › Supplementary Table S5_Combined Haplotype.pdf]

Supplementary Table S5. Number of materials that have the haplotype of resistance/susceptibility to SNPs Chr06:49886965 (T/C) and Chr18:1872252 (T/G), per phenotypic classes, identified in the mapping for the isolate IBS 333 and IBS 327, respectively. Materials displaying heterozygosity, those exhibiting contrasting phenotypes, or those evaluated solely for one isolate were excluded from the analysis.

Haplotype ID	Positions in the soybean genome		*X. citri* reaction (IBS 333 and IBS 327)			
	Chr06:49886965	Chr18:1872252	I/R/MR*	MS/S/HS	n	Accuracy
Hap-resistant	T	T	34	2	36	94%
Hap-susceptible	C	G	2	34**	36	94%
Hap-alternative1	T	G	12	4	16	
Hap-alternative2	C	T	13	14	27	
						**115**

* 25 of these materials reacted as immune to isolate IBS 327

** 1 of these materials reacted as highly susceptible to both isolates
